# The Changes of Histone Methylation Induced by Adolescent Social Stress Regulate the Resting-State Activity in mPFC

**DOI:** 10.34133/research.0264

**Published:** 2023-10-31

**Authors:** Jiesi Wang, Wei Zhang, Hang Xu, Bart Ellenbroek, Jiajie Dai, Li Wang, Chaogan Yan, Weiwen Wang

**Affiliations:** ^1^ CAS Key Laboratory of Mental Health, Institute of Psychology, Chinese Academy of Sciences, Beijing, China.; ^2^ Department of Psychology, University of Chinese Academy of Sciences, Beijing, China.; ^3^ School of Psychology, Victoria University of Wellington, Kelburn, Wellington 6012, New Zealand.; ^4^ CAS Key Laboratory of Behavioral Science, Institute of Psychology, Chinese Academy of Sciences, Beijing, China.

## Abstract

Early-life stress can lead to sustained alterations in regional resting-state brain functions, but the underlying molecular mechanism remains unclear. Stress can also induce sustained changes in epigenetic modifications across brain regions, which are not limited to a few genes; rather, they often tend to produce global levels of change. The functional implication of these changes also remains to be elucidated. We hypothesize that global epigenetic changes may partly modulate the resting-state functions of brain regions to influence behavior. To test this hypothesis, we used an adolescent social stress (ASS) model in mice and examined the relationship between epigenetic modifications and regional resting-state brain activity using resting-state functional magnetic resonance imaging (rs-fMRI). The results showed that, compared to the control mice, the stressed mice showed increased anxiety and social avoidance behaviors and greater levels of dimethylation of histone H3 at lysine 9 (H3K9me2) in the medial prefrontal cortex (mPFC). In addition, the resting-state activity represented by the amplitude of low-frequency fluctuation (ALFF) was significantly lower in the mPFC of stressed mice. To verify the relationship of H3K9me2 and ALFF, the specific inhibition of H3Kme2 was performed by using the drug UNC0642, which reversed the anxiety behavior induced by ASS and significantly increase the ALFF value of mPFC in both normal and ASS animals. Our study is the first to report an association between histone modifications and rs-fMRI findings, providing a new perspective for understanding of the significance of regional brain epigenetic changes and a possible molecular explanation for rs-fMRI findings.

## Introduction

Stress, especially during early life, can lead to persistent, even lifelong, alterations in brain structures and functions that affect multiple behavioral domains [1]. The brain functional changes caused by early stress are evident not only in abnormal brain responses during behavioral tasks but also in the changes in regional intrinsic activity and functional network during the resting state [2–5]. However, the functional significance and biological basis of these resting-state changes, which are detected using resting-state functional magnetic resonance imaging (rs-fMRI) based on blood oxygen level-dependent (BOLD) signal, remain unclear. One important reason is that the resting-state BOLD signal is a reflection of the synchronized neuronal activity in the low-frequency (typically 0.01 to 0.1 Hz) [6], but which mechanisms of synchronization in neural cells that underlie it are still unknown.

Cellular activities in tissue are synchronized at many levels. One of the interesting ones, at the molecular level, is the synchronization of epigenetic modifications, such as DNA methylation or histone modifications, between cells. The synchronized changes of epigenetic modifications are not only present in a few genes but are often accompanied by convergent changes in multiple other genes due to nonspecific regulated enzymes [7,8]. This phenomenon is especially evident in the nervous system, where stress can lead to a global induction or suppression of particular epigenetic modifications in certain brain regions [9–11], resulting in convergent transcriptional changes of thousands of genes. The functional significance of these global epigenetic changes remains unclear, but they have been shown to affect various behaviors in animals [12–15]. Interestingly, such regionally global epigenetic changes could be identified as a manifestation of the synchronized changes of regional nerve cells at the transcriptome level under a “resting state” [16]. Therefore, we hypothesize that the global changes of epigenetic modifications may be one of the molecular mechanisms underlying rs-fMRI changes.

To validate this hypothesis, an early adolescent social stress (ASS) model was chosen because previous studies by our group and others have shown that ASS can lead to a series of persistent behavioral abnormalities and alter the expression of a histone methylation marker, H3K9 dimethylation (H3K9me2), in the brain-derived neurotrophic factor (BDNF) promoter region in the medial prefrontal cortex (mPFC) in adult mice [17]. The mPFC region plays critical roles in negative emotions, cognitive abilities, and social behaviors [18,19], which can also be regulated by multiple epigenetic modifications, including global-level alterations [20,21]. Therefore, we further examined whether global H3K9 methylation was also effected by ASS in this brain region. After confirming the validity of the ASS model, we performed rs-fMRI and then collected samples of different brain regions for measuring global histone methylation levels. We used the amplitude of low-frequency fluctuation (ALFF), which measured fluctuations of resting-state BOLD signals, as the main indicator in rs-fMRI analysis because it has high stability and reproducibility in repeated measurements [22] and clearer biological significance [23]. Moreover, we explored the associations among histone methylation, rs-fMRI measures, and behavioral phenotypes by linear regression analysis and specific inhibition of histone methylation.

## Results

### The behavioral changes in the ASS model

**Fig. 1.**
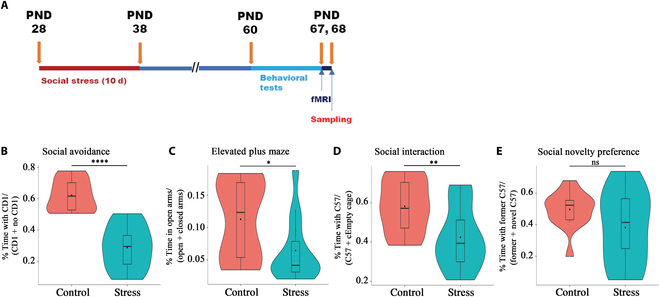
The changes of behaviors in the ASS model. (A) Schematic timeline (PND, postnatal days; behavior tests are elevated plus maze test, social avoidance test, and 3-chamber test, performed in this order). (B) Social interaction ratio in the social avoidance test. (C) Open-arm ratio in the elevated plus maze test. (D) Social exploration ratio in the 3-chamber test. (E) Novel social preference in the 3-chamber test (*P < 0.05, **P < 0.01, ****P < 0.001; *t* test; control versus stress; *n* = 14 or 13).

The schematic timeline of the ASS model, rs-fMRI scanning, and sampling is shown in Fig. 1A. Animals that experienced ASS showed a series of behavioral abnormalities in adulthood compared with control mice (Fig. 1B to E), including the lower social-interaction ratio in the social avoidance test (*t* = 7.20, *P* < 0.001, *R*^2^ = 0.67; Fig. 1B), the lower open-arm ratio in the elevated plus maze test (*t* = 2.25, *P* < 0.05, *R*^2^ = 0.17; Fig. 1C), and the decreased social exploration ratio in the 3-chamber social interaction test (*t* = 2.80, *P* < 0.01, *R*^2^ = 0.24; Fig. 1D), but the novel social preference score was not significantly different between the groups (*P* > 0.05; Fig. 1E). More raw data of these behavior tests are shown in Fig S1. These results indicated that ASS can increase social avoidance and anxiety-like behaviors and decrease the general social interest in the adult mice.

### The changes of histone H3K9 methylation and resting-state activity in mPFC in the ASS model

**Fig. 2.**
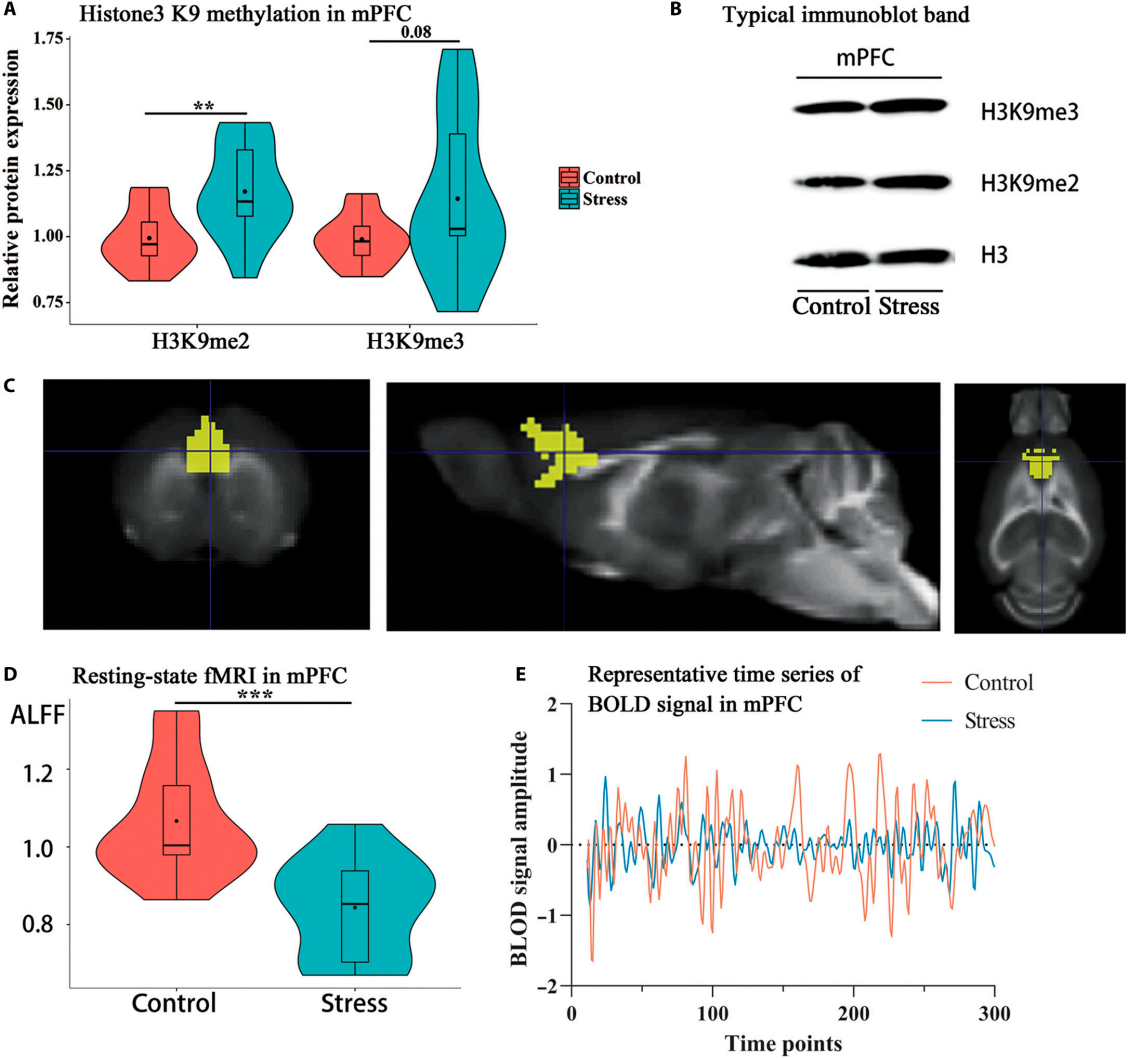
The association of ALFF values and H3K9 methylations levels in mPFC in the ASS model. (A) Levels of H3K9me2 and H3K9me3 in the mPFC (**P < 0.01; *t* test; control versus stress; *n* = 14 or 13). (B) Typical immunoblot band of histone H3K9me2. (C) The ROI of the mPFC is shown on the template. (D) Extracted data for the ALFF in the mPFC (***P < 0.001; *t* test; control versus stress; *n* = 14 or 13). (E) Representative time series of BOLD signal in mPFC (0.01 to 0.1 Hz filtered).

Our previous study found that the H3K9 dimethylation level in the promoter region of the BDNF gene in the mPFC was significantly increased after ASS. Accordingly, we measured the global levels of H3K9me2 and H3K9me3 in the mPFC. The results showed that the levels of H3K9me2 and H3K9me3 were increased in the mPFC in the stress (STR) group compared with the control (CON) group; the increase in H3K9me2 was significant (*t* = 3.11, *P* < 0.01, *R*^2^ = 0.28; Fig. 2A and B), while the increase in H3K9me3 was almost significant (*t* = 1.81, *P* = 0.0829, *R*^2^ = 0.12; Fig. 2A and B). The 2 modifications in the anterior agranular insular cortex (AIa), an area in close proximity to the mPFC and also considered to be associated with negative emotions and cognition [24,25], did not show significant differences between groups (*P* > 0.05; Fig. S2), which indicated that the changes in histone modification were specific to the mPFC. In addition, the results of rs-fMRI showed that the ALFF levels in the mPFC were significantly lower in the STR group than in the CON group (*t* = 4.21, *P* < 0.001, *R*^2^ = 0.42, Fig. 2C to E). Further linear regression analysis of ALFF on the levels of H3K9me2 or H3K9me3, conducted separately within each group, did not show any significant correlations (all *P* > 0.05).

**Fig. 3.**
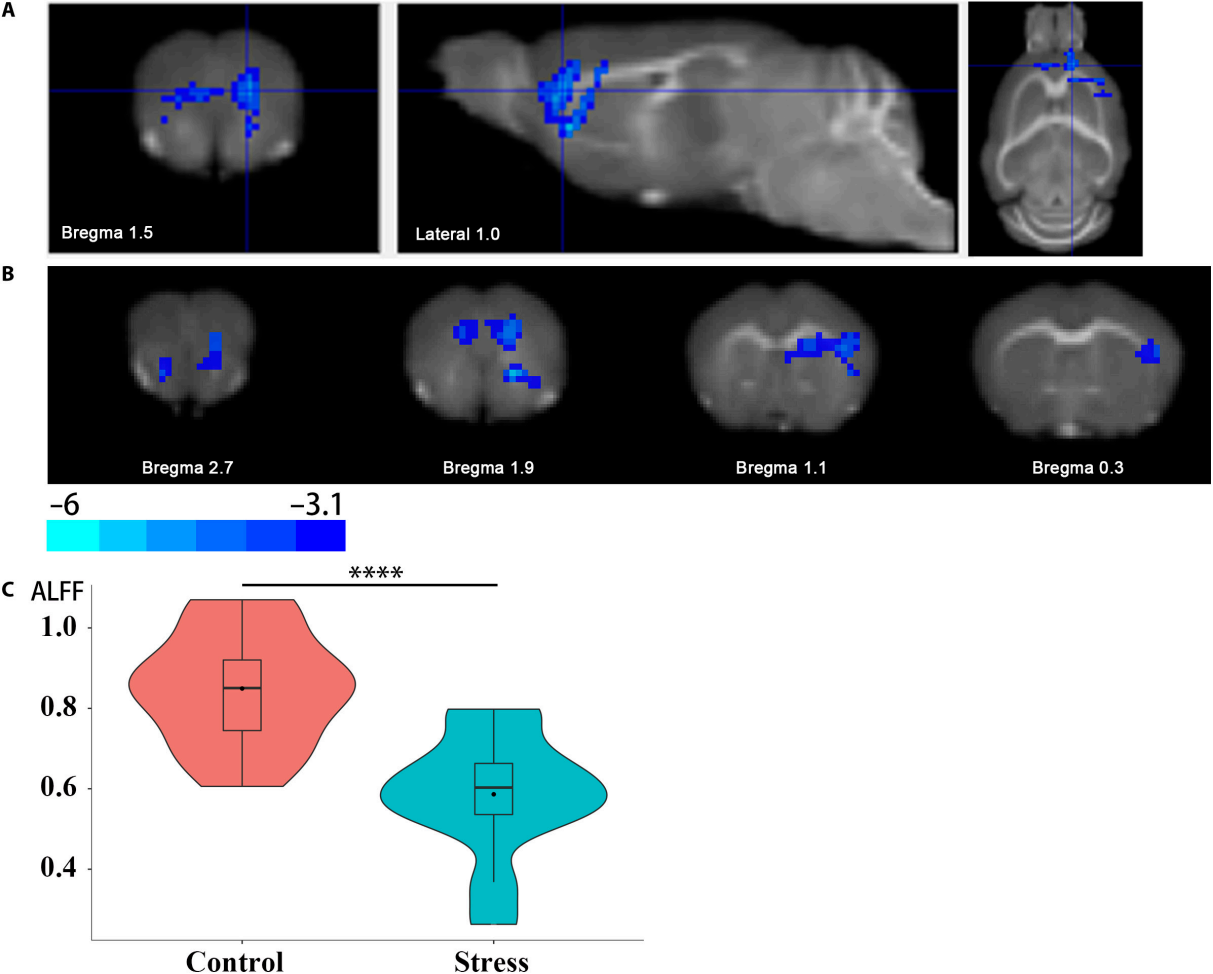
Whole-brain voxel-based analysis of ALFF values. (A and B) ALFF values in the front of the brain (mainly in the mPFC) were significantly decreased in stressed mice compared to control mice (voxel *P* < 0.001, cluster *P* < 0.05, GRF correction). (C) Extracted data for the ALFF of the significant cluster (*t* test, ****P < 0.001, control versus stress; *n* = 14 or 13).

Further whole-brain voxel-based analysis was performed based on ALFF values. After multiple comparison correction (voxel *P* < 0.001, cluster *P* < 0.05, Gaussian random field [GRF] correction), only one cluster was found around the mPFC region (Fig. 3A and B), and the ALFF in this region was significantly lower in mice that were exposed to ASS than in CON mice (*t* = 4.66, *P* < 0.001, *R*^2^ = 0.47; Fig. 3C), which indicates that the changes of ALFF values in mPFC may be the most significant.

Furthermore, a whole-brain seed-based analysis of functional connectivity was performed by using an mPFC template as the seed, but no brain regions were found a significant difference between ASS and control mice after multiple comparison correction (voxel *P* < 0.001, cluster *P* < 0.05, GRF correction). Specific extraction of the functional connectivity between mPFC and 3 subregions of the amygdala (basal, central, media), as a previous study showed these connections may associate with early stress [26], was also performed; no significant difference was found between groups (Fig. S3).

### The effect of histone K9 dimethylation inhibition on the resting-state activity of mPFC of normal mice

**Fig. 4.**
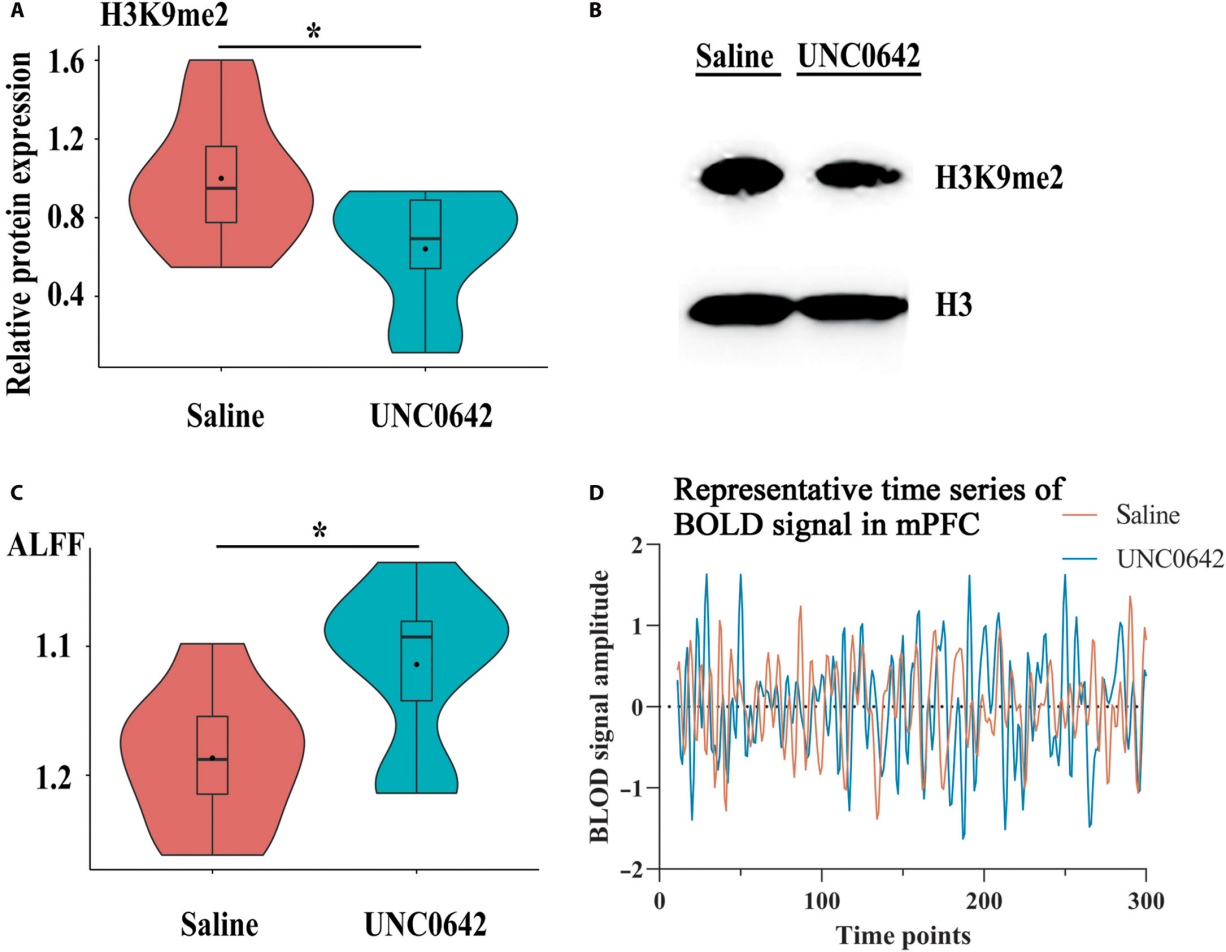
The inhibition of H3K9me2 increased ALFF value in mPFC. (A) Levels of H3K9me2 in the mPFC (*P < 0.05; *t* test; saline versus UNC0642; *n* = 8). (B) Typical immunoblot band of H3K9me2. (C) Extracted data for the ALFF in the mPFC (*P < 0.05; *t* test; saline versus UNC0642; *n* = 8). (D) Representative time series of BOLD signal in mPFC (0.01 to 0.1 Hz filtered).

For validation of the association of H3K9me2 and ALFF, firstly, normal adult mice were treated with UNC0642, a G9a-GLP heteromeric complex (H3K9me2 methylases) inhibitor. The intervention of H3K9me2 levels on unstressed normal mice allows us to validate the relationship between H3K9me2 and ALFF values only while avoiding the other variables introduced by stress. The results showed that, compared with the saline group, the administration of UNC0642 significantly decreased the level of H3K9me2 (*t* = 2.22, *P* < 0.05, *R*^2^ = 0.26; Fig. 4A and B) and increased the ALFF value (*t* = 2.49, *P* < 0.05, *R*^2^ = 0.31; Fig. 4C and D) in mPFC. Further linear regression analysis in separate group also did not find significant associations between H3K9me2 and ALFF within each group (all *P* > 0.05). Moreover, this drug treatment did not affect the level of H3K9me3 (Fig. S4), which indicated that the drug intervention was specific to the H3K9me2 in vivo.

### The effect of histone K9 dimethylation inhibition on behaviors and the resting-state activity of mPFC in the ASS model

**Fig. 5.**
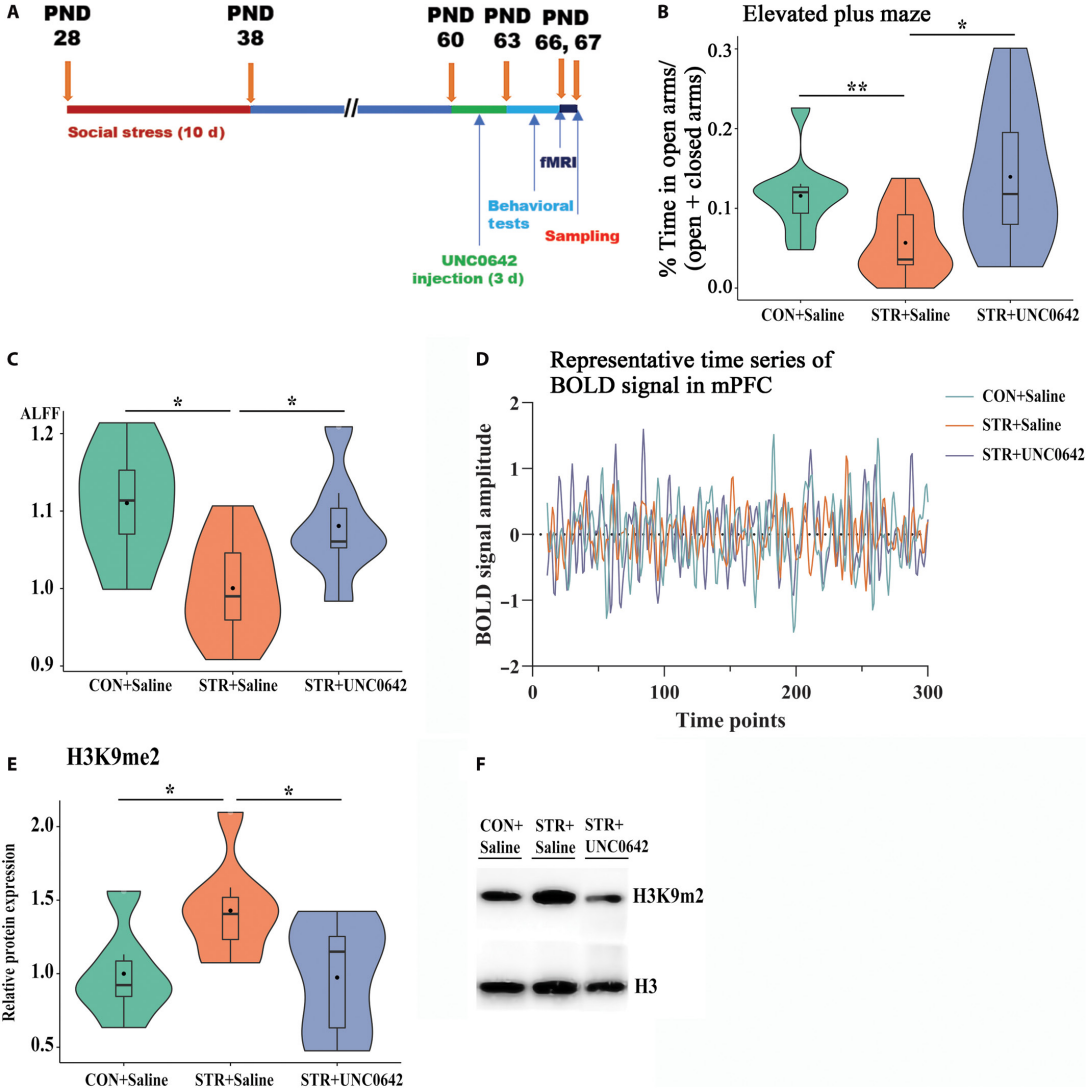
The inhibition of H3K9me2 reversed the increase of anxiety and the reduction of ALFF value in mPFC. (A) Schematic timeline. (B) The open-arm ratio after the administration of UNC0642 (*P < 0.05; one-way ANOVA; *n* = 9 to 10). (C) Extracted data for the ALFF in the mPFC. (D) Representative time series of BOLD signal in mPFC (0.01 to 0.1 Hz filtered) (*P < 0.05; one-way ANOVA; *n* = 9 to 10). (E) Levels of H3K9me2 in the mPFC (*P < 0.05; one-way ANOVA; *n* = 6 to 8). (F) Typical immunoblot band of H3K9me2.

For further validation of the associations of histone H3K9 methylation with resting-state activity changes and behavioral abnormalities, the UNC0642 was used in the ASS model, and the schematic timeline was shown in Fig. 5A. After the UNC0642 administration, the analysis of variance (ANOVA) analysis showed that the drug treatment reversed the anxiety behavior (*F* = 4.43, *P* < 0.05, *R*^2^ = 0.25; Fig. 5B) but did not affect the social avoidance (Fig. S4). In addition, UNC0642 significantly affected the ALFF value (*F* = 6.38, *R*^2^ = 0.33; Fig. 5C and D) and the level of H3K9me2 (*F* = 3.82, *P* < 0.05, *R*^2^ = 0.31; Fig. 5E and F) in mPFC. The post hoc analysis indicated that the ASS significantly increased the anxiety behavior and the level of H3K9me2 of mPFC and decreased the ALFF value of mPFC (all *P* < 0.05, CON+Saline versus STR+Saline); the administration of UNC0642 significantly reversed these changes (all *P* < 0.05, STR+UNC0642 versus STR+Saline).

## Discussion

In the present study, the histone modifications H3K9me2 were increased in the mPFC, and resting-state brain activity, indexed by the ALFF, was decreased in the same brain region. Regression analyses showed that the H3K9me2 levels and the ALFF value in the mPFC were significantly associated. The inhibition of H3K9me2 reversed the increase of anxiety behavior induced by ASS; meanwhile, it also increased the ALFF value in the mPFC of both stressed and unstressed normal mice. To our knowledge, our study is the first to link epigenetic mechanisms with rs-fMRI findings.

Our results demonstrated that ASS causes long-term decreases in ALFF levels in the mPFC regions. Additionally, the whole-brain voxel analysis revealed that ASS reduced the ALFF values around the mPFC region compared with the control group. However, no significant changes in functional connectivity between mPFC and other brain regions were observed. These results are consistent with previous studies that have demonstrated that adolescence is a sensitive period during which stress exposure can affect the structure and function of the mPFC [27,28]. Our study provides further evidence that ASS can decrease the intrinsic activity of the mPFC. Furthermore, the consistent changes of ALFF values and anxiety-like behavior in ASS models and H3K9me2 intervention indicate that the early-stress induced changes of the intrinsic activity of mPFC may play a key role in anxiety behaviors.

The most important finding of this study was that it linked epigenetic mechanism with rs-fMRI findings. In a previous study, we found that ASS can induce persistent increases in H3K9me2 in the promotor region of the BDNF gene in the mPFC [17]. However, the current results show that this increase is not limited to specific genes; it also occurs at the global level in this region. Most previous studies have explained these kinds of global epigenetic modification changes based on the functions of the down-regulated genes [15,16]. Our results provide the first integrated evidence showing that global increases in H3K9me2 in the mPFC may regulate the resting-state intrinsic activity of this region. The drug UNC0642, a highly selective and brain-permeable inhibitor of G9a/GLP (H3K9me2 methylase) [29,30], was used to verify this relationship by specifically reducing the H3K9me2 levels of mPFC in both stressed and normal animals. The results showed a consistent negative association between H3K9me2 levels and ALFF values across all the patches of animals.

This negative association may result from the influence of histone modification on energy metabolism in the brain. Rs-fMRI is a technology based on the use of BOLD contrast, which reflects neuronal energy consumption. Intriguingly, most of the brain’s energy is consumed at rest, and the difference in energy consumption between states of brain activity and the resting state is quite small [31]. Therefore, rs-fMRI is very closely associated with brain energy metabolism, which may be affected by H3K9m2 in 2 ways. First, the changes in gene transcription induced by global H3K9me2 may directly lead to a detectable disturbance of regional energy consumption. H3K9me2 is a kind of negative epigenetic modification; increases in H3K9me2 cause chromosomes to be less accessible to regulatory factors, which in turn down-regulates the expression of genes [32]. Therefore, the increase of global H3K9me2 can reduce the energy consumption for the transcription and the protein synthesis of abundant genes in a mass of cells, which may be directly reflected in the BOLD contrast. Second, the H3K9me2 may regulate some key processes of neuronal energy consumption. The energy-consuming processes in the brain can be broadly divided into 2 fractions: signaling processes and nonsignaling processes [33]. The former may account for more than 60% of the total consumption and is used to support cellular activities such as neuronal firing or γ-aminobutyric acid (GABA)/glutamate cycling, while the latter is used to support cellular activities such as protein and lipid synthesis or glial motility [33,34]. Krishnan et al. [35] indicated that the brain’s resting-state activity is associated with Na+/K+ pumps, AMPA/GABA synaptic currents, and glial properties. Interestingly, lots of the cellular processes mentioned above have been found to be regulated by H3K9 methylation. For example, the K+ channel can be suppressed by H3K9me2-modulated silencing of related genes [36], and early stress-induced abnormalities of microglia have also been associated with H3K9me2 [37]. Moreover, the gene mecp2, which plays important roles in GABAergic neuronal functions [38], can regulate H3K9 methylation states in chromosomes [39]. Therefore, global changes of H3K9me2 may simultaneously affect multiple such cellular processes, which are then reflected in the BOLD signal. Further investigations are needed to verify these 2 hypotheses.

Linking epigenetic mechanisms with brain resting-state functions expands the biological significance of the findings in the both fields. In the epigenetic field, many epigenetic studies have focused on identifying the genes or pathways that can be regulated by epigenetic modifications, but few studies have explored the role of epigenetic mechanisms in synchronizing intercellular transcriptional activity. Our results suggest that specific epigenetic modifications may coordinate the activity of cells that contribute to regional brain functions, which further implies that epigenetics may also regulate brain networks or brain homeostasis, which are common topics in rs-fMRI studies. Previous research has examined the effects of epigenetic changes on brain networks and neuronal connectivity [40], but resting-state functional connectivity has received less attention. From the perspective of brain resting-state functions, our results propose a novel potential molecular mechanism underlying rs-fMRI findings, which will offer new insights for the molecular investigation based on rs-fMRI results.

The present study has some limitations that should be acknowledged. First, the sample size of this study was smaller than that of most human brain imaging studies because of experimental constraints. However, we believe that the sample size was adequate for the present study because the C57 mice used had the same genetic background, and environmental factors were strictly controlled during the experiment. Second, based on a previous study [17], we tested 2 histone modifications, H3K9me2 and H3K9me3. On the one hand, this choice was made to avoid the investigation of a variety of epigenetic modifications, which would have complicated the investigation, as complex interactions exist among different modifications. On the other hand, the potential contributions of other modifications to resting-state activity need to be investigated in the future to provide more information. Third, we used systemic administration via intraperitoneal injection for the drug intervention experiment in this study, which avoided the perturbations to the fMRI scanning by implanted cannulas of stereotactic injection but also reduced the regional specificity of the effect of H3K9me2 inhibition on behavior and brain function. In the future study, other alternative approaches such as using an adeno-associated virus combined with G9a-specific short hairpin RNA to regionally knock down the G9a mRNA expression can be used to enhance regulatory specificity. Fourth, as the challenging to conduct ASS in female mice, the current study was performed with only male mice and in the absence of consideration for sex difference. Last, although this study intervened and validated the relationship between histone methylation and resting-state brain activity through specific inhibitors, it still belongs to association research. Additionally, despite observing synchronous changes in H3K9me2 and ALFF with stress and drug intervention, their association was not significant in separate within-group regression analyses. The causal relationship between the 2 factors still needs to be confirmed by more fundamental and specific methods in further studies.

In conclusion, this study is the first to report a relationship between resting-state activity in the mPFC and behavioral changes in adult mice exposed to ASS. Moreover, this study reveals that regional global histone methylation regulated changes in brain resting-state activity and suggests that epigenetic mechanisms may underlie rs-fMRI findings in the brain.

## Materials and Methods

### Animals

Male C57BL/6J mice were used in the experiments. The mice were bred in our animal facility, and their parental mice were purchased from the Vital River Laboratory Animal Center (Beijing, China). The mice were exposed to ASS on postnatal day (PND) 28. To establish ASS, 14 aggressive CD1 mice (Vital River) were screened as residents. All animals were housed under standard conditions (12-h light/dark cycle; temperature, 22 ± 1 °C; humidity, 55% ± 10%) and given free access to food and water. All experiments were performed in accordance with the Guidelines for the Care and Use of Laboratory Animals of the Chinese Academy of Sciences.

### ASS

ASS was elicited as in our previous studies [17,41]. First, 14 adult aggressive CD1 male mice (12 to 20 wk old) were screened, and these mice had been kept in a single cage for more than 1 month in advance to enhance the aggressiveness; the standard for selection was an attack latency for a CD1 mouse against a C57 (PND28) mouse of less than 1 min on 3 consecutive days. The PND28 C57 mice were separated into 2 groups: a STR group and a CON group. During the 10-d stress period, each STR mouse was introduced into a novel cage, exposed to a different CD1 mouse for 5 min each day, separated from the CD1 mouse by a clear perforated Plexiglas divider, and then housed together with the CD1 mouse (with the divider in place) for 24 h. Each CON mouse was exposed to a novel C57 mouse each day under the same paradigm but was separated from the C57 mouse with no physical contact. The schematic timeline is shown in Fig. 1A.

### Drug administration

UNC0642 (Selleck, Houston, USA), a potent, selective inhibitor of histone methyltransferases G9a/GLP (also named EHMT1 and EHMT2), was dissolved in dimethyl sulfoxide stoke, and diluted with saline before use (dimethyl sulfoxide = 0.2% W/V in working solution). The drug was systemically administrated to the animals via intraperitoneal injection (1 mg/kg) for 3 d before fMRI scanning, and the dosage and frequency of the administration are based on a previous study [42]. For normal mice, adult animals were tested for social avoidance behavior after the final drug administration and performed fMRI scanning and tissue extraction after the behavior test. For ASS mice, the schematic timeline is shown in Fig. 5A; the baseline of social avoidance was tested on PND60, and then the stressed mice were divided into 2 groups to administrate with UNC0642 or saline. The procedure of administration is the same as the normal mice. The control mice were given saline in the same procedure.

### Behavioral tests

The behavioral tests were performed starting on PND60 in the order described. All test animals were placed in the test room for 1 h before each test began, and sequential behavioral tests were separated by 48 h.

#### Social avoidance test

The procedure for social avoidance testing was based on a previous study [43]. The test was performed in a square white plastic chamber (*L* × *W* × *H*: 40 × 40 × 20 cm). A fixed metal cage (*L* × *W* × *H*: 10 × 5 × 20 cm) was placed at the center beside one of the chamber walls. The 8-cm range around the metal cage was defined as the social interaction area.

The test was divided into 2 sessions. In the first session, a C57 mouse was placed in the test chamber for free exposure for 2.5 min. In the second session, a strange CD1 mouse was placed in the metal cage, and the C57 mouse has introduced into the chamber again for another 2.5 min. During the sessions, the mice were monitored with a camera, and the amount of time each mouse stayed in the interaction area was recorded automatically by software (Anilab, Ningbo, China). All sessions were performed under totally black conditions, and the test cage was carefully cleaned with 75% alcohol between test mice.

Mouse social avoidance behavior was represented by the social interaction ratio, which was calculated as follows:

$$\begin{array}{c} \mathrm{social interaction ratio}=\text{time spent with CD1}\;\mathrm{mouse in second session}/\\ \left(\mathrm{time spent with CD1}\;\mathrm{mouse in second session}+\mathrm{without CD1}\;\mathrm{mouse in first session}\right)\times \text{100}\%. \end{array}$$

We calculated the social interaction ratio as the above instead of as the time spent with CD1/without CD1, which is more commonly used in previous studies, because the present ratio is linear and more suitable for further linear regression analysis.

#### Elevated plus maze test

The test procedure for the elevated plus maze was based on previous research [44]. When the test was initiated, each mouse was placed into the middle area with its head facing the open arms. During the 10-min test, the mouse was free to move, and its activity and the amount of time it stayed in each area were automatically tracked and recorded by the software. The test was performed under dim-light conditions; after each mouse test was completed, the entire maze was cleaned with 75% alcohol. The open-arm ratio was chosen as the main parameter in this test and was calculated as follows:

$$\begin{array}{c} \text{open}-\mathrm{arm}\;\text{ratio}=\text{time spent in open}\;\mathrm{arm}/\\ \left(\text{time spent in open}\;\mathrm{arm}+\text{closed}\;\mathrm{arm}\right)\times \text{100}\%. \end{array}$$

#### Three-chamber social test

A 3-chamber test was carried out with reference to a previous study [45]. The test cage (*L* × *W* × *H*: 60 × 40 × 25 cm) was made of white plastic and divided into 3 equal-area chambers (*L* × *W* × *H*: 20 × 40 × 25 cm) by a transparent divider with an 8 × 8 cm door at the center of the bottom. Two metal cages (*L* × *W* × *H*: 10 × 10 × 25 cm) were placed in the 2 outermost side chambers to enable the introduction of social objects during the test. The test had 3 sessions. In the first session, the doors on the divider were closed; the mouse was placed in the middle chamber, allowed to freely explore for 5 min, and then returned to its home cage. In the second session, the doors were opened, and a male C57 mouse was placed in one of the 2 metal cages; the chamber in which the mouse was located was defined as the social area, and the opposite chamber was defined as the blank area. The test mouse was then placed in the middle chamber and allowed to explore the 3 chambers freely for 10 min. In the third session, a novel male C57 mouse was placed in an empty metal cage before the start of the test, and the area was defined as the novel social area. The other metal cage remained unchanged, and the surrounding chamber was defined as the old social area. The test mouse was then placed in the chambers again for 10 min. In all sessions, the residence times of the mouse in the 3 chambers were recorded by software (Anilab). Two parameters were analyzed in this test and calculated as follows:

$$\begin{array}{c} \text{social exploration ratio}=\text{time spent in social area}/\\ \left(\text{time spent in social area}+\text{blank area}\right)\times \text{100}\% \end{array}$$

$$\begin{array}{c} \text{novel social preference}=\text{time spent in novel social area}/\\ \left(\text{time spent in novel social area}+\mathrm{old}\;\text{social area}\right)\times \text{100}\%. \end{array}$$

### Rs-MRI

#### Image acquisition

All data were obtained by a 7T MR scanner (Bruker, UK) at the Institute of Materia Medica, Chinese Academy of Medical Science. During the scanning process, the mice were anesthetized under 1% to 2% isoflurane with oxygen as the carrier gas. The anesthesia concentration was adjusted in real time according to the mouse breathing and heart rate detection system; the heart rate was maintained at 60 to 80 beats per minute to ensure that the degree of anesthesia was light. Meanwhile, a water bath insulation system was used to keep the body temperature of mice stable during the anesthesia. A routine T2-weighted image was acquired using the following parameters: image size = 256 × 256, field of view = 20 × 20 mm, slice thickness = 0.4 mm, number of slices = 28, and echo time/repetition time = 35/3,150 ms. Then, 2 resting-state BOLD image sessions were performed using the following parameters: image size = 64 × 64, field of view = 20 × 20 mm, slice thickness = 0.4 mm, number of slices = 28, echo time/repetition time = 15/2,000 ms, and repetitions = 300.

#### Image preprocessing

Preprocessing was performed with the rat module of the Data Processing Assistant for Resting-State fMRI (DPARSF) V4.1 in DPABI V4.1 [46]. Briefly, the voxel dimensions were augmented 10 times to adapt the software, which was designed for human imaging. The first 10 time points were removed, and the slice-timing correction was performed. Caret map_015, an MRI-based and distortion-free mouse template, was used as the T1 template, which has been used in previous studies [47]. The anatomical images of each animal were normalized to this template, and then the same parameters were applied to the functional images. After realignment, the 6 head motion parameters were regressed out from the functional data to minimize the influence of nuisance covariates. The mALFF was analyzed, and the data were smoothed with a full-width half-maximum kernel at 0.6 mm. A 0.01- to 0.1-Hz band-pass filter was used for the functional data. Global signal regression was not performed. Finally, the mALFF data from the 2 scan sessions were averaged. The templates for the regions of interest (ROIs) were supplied by Dr. D. Fair and were based on the Allen Database; they have been publicized previously [47]. In the present study, the mPFC brain region, composed of the prelimbic cortex and infralimbic cortex ROIs, was chosen as the main ROI.

### Tissue extraction

After all the tests were completed, the mice were sacrificed by carbon dioxide. Their entire brains were quickly extracted, frozen in liquid nitrogen, and then stored in a −80 °C freezer until use. The tissue of the mPFC and anterior AIa was punched with a needle of 1-mm inner diameter in a freezing microtome. The AIa is an area that has also been considered to be closely correlated with negative emotions and cognitive abilities [24,25], and it is not far from the mPFC; it was chosen to identify the regional specificity of histone methylation. The extraction site was chosen with reference to the mouse brain atlas, and the detailed locations are shown in Fig. 1. The collected tissues were quickly placed in precooled tubes and stored in a −80 °C freezer.

### Western blot analysis

The protocol for western blot analysis was similar to that used in our previous studies [41]. Tissue protein was extracted using radio-immunoprecipitation assay lysis buffer (CW233s, CWBIO, Beijing, China) with a cocktail of protease inhibitors (Roche, Mannheim, Germany) according to the manufacturer’s protocol. The extracted protein was quantified using a BCA Protein Assay kit (CW0014, CWBIO), mixed with reducing 5× protein loading buffer (CW0027S, CWBIO), and denatured. The protein samples were separated by sodium dodecyl sulfate-polyacrylamide gel electrophoresis in reducing conditions using 12% acrylamide gels (Millipore, Bedford, MA, USA). After membrane transfer and blocking, the membranes were incubated with anti-rabbit H3K9 trimethylation (H3K9me2) (1:1,000; 4658, Cell Signaling Technology, MA, USA) primary antibodies. After the following secondary antibody incubation, the signals were detected using the enhanced chemiluminescence method and photographed. Then, the membranes were stripped of protein twice with antibody stripping buffer, and it was confirmed that no detectable signal could be observed after stripping. The membranes were then incubated again with anti-mouse H3 antibodies (1:1,000; 14269, Cell Signaling Technology), and processes similar to those previously described were performed. After that, the membranes were stripped again and incubated with anti-rabbit H3K9me3 antibodies (1:1,000; 13969, Cell Signaling Technology) to detect another signal. The band intensities of H3K9me3 and H3K9me2 were semiquantitatively analyzed by normalization to the intensity of H3 using Quantity One (Bio-Rad, USA). The samples from the 2 groups were equally allocated to 2 gels, and all the procedures for the samples were performed at the same time. There were no significant differences in the intensities of samples in the same group between the 2 gels.

### Statistics

For the fMRI results, DPABI V4.1 was used to extract the mALFF data and time series of the ROIs, as well as perform voxel-based whole-brain statistical analysis. Other statistical methods were completed using GraphPad Prism 8. Intergroup comparison data were presented as a combination of box and violin plots, drawn using the ggplot2 package in R. The unpaired *t* test was used to test differences between 2 groups, while one-way ANOVA with false discovery rate (Benjamini and Hochberg method) post hoc test was used for 3 groups. The linear regression of histone methylation and ALFF was performed within each group separately and using histone methylation levels as independent variables. The Brown–Forsythe test confirmed no significant difference in variance between groups (*P* > 0.05) in *t* test or one-way ANOVA. The Gaussian distribution of residuals in all statistical analyses was confirmed using the Anderson–Darling test (all *P* > 0.05). The statistical significance threshold for all analyses was *P* ≤ 0.05, and the effect size was reported using R square.

## Data Availability

All data needed to evaluate the conclusions in the paper are present in the paper and/or the Supplementary Materials. More data that support the findings of this publication are available upon request from the corresponding author.
