# Supplementary figures and images for: The Changes of Histone Methylation Induced by Adolescent Social Stress Regulate the Resting-State Activity in mPFC
Source: Research (Wash D C). 2023 Oct 31;6:0264. doi: 10.34133/research.0264 (PMC10907022; doi:10.34133/research.0264)

## Social Avoidance

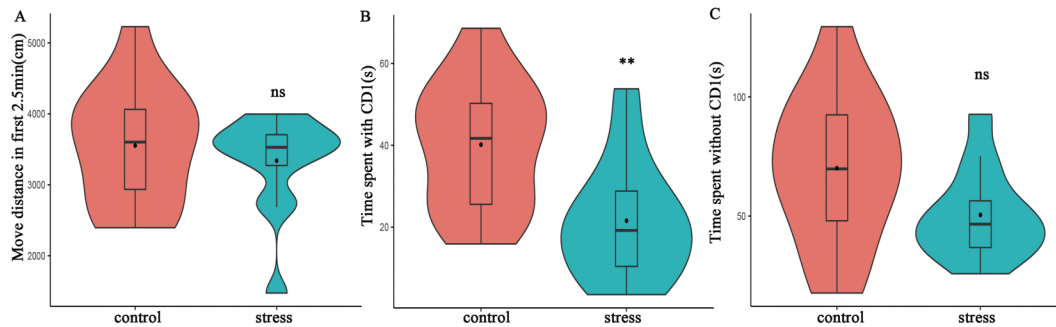

## Elevated Plus Maze

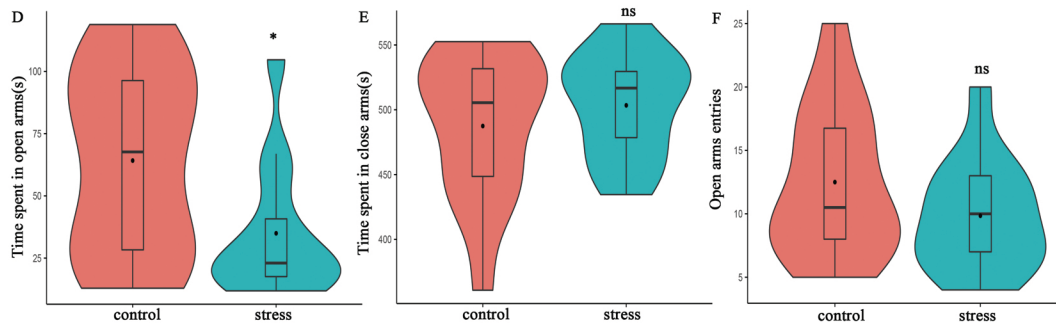

## 3-chamber Test

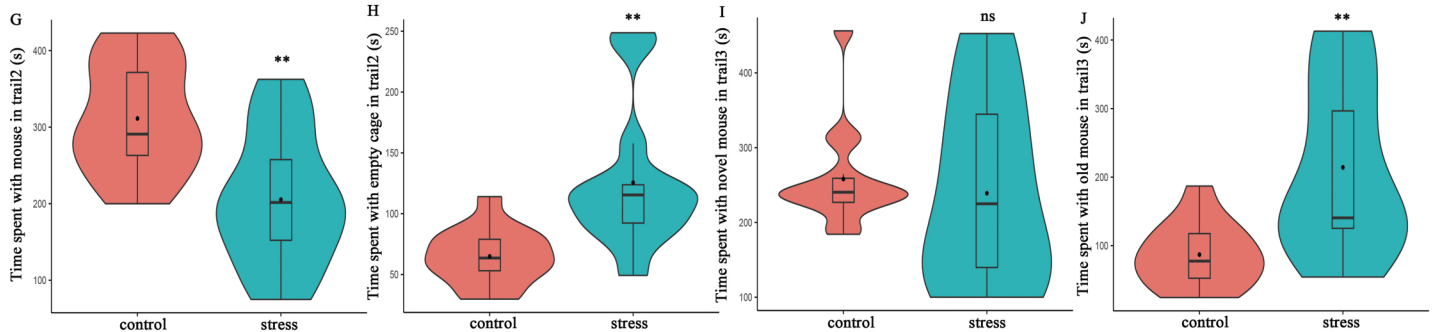

Supplement: Supplementary 1 — Figs. S1 to S4 [file research.0264.f1.zip › Figure S1.pdf]

**A****Ala**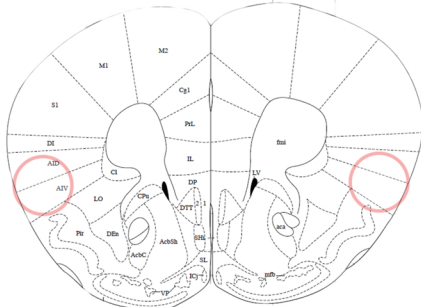**Bregma 1.78**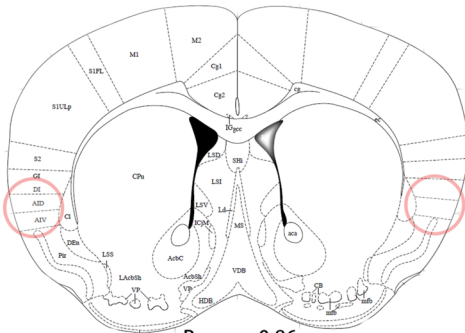**Bregma 0.86****mPFC**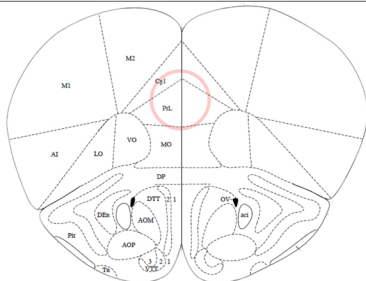**Bregma 2.10**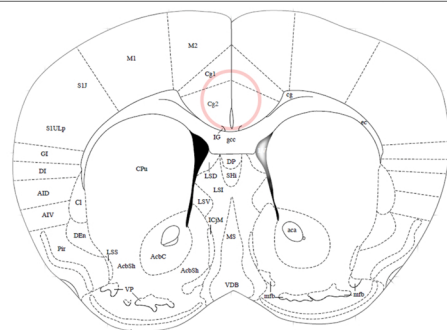**Bregma 1.10****B****Ala**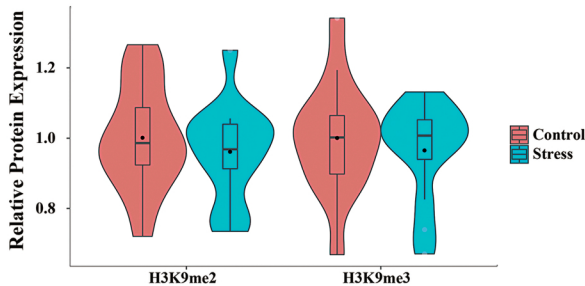**C Typical immunoblot band**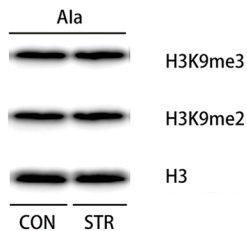

Supplement: Supplementary 1 — Figs. S1 to S4 [file research.0264.f1.zip › Figure S2.pdf]

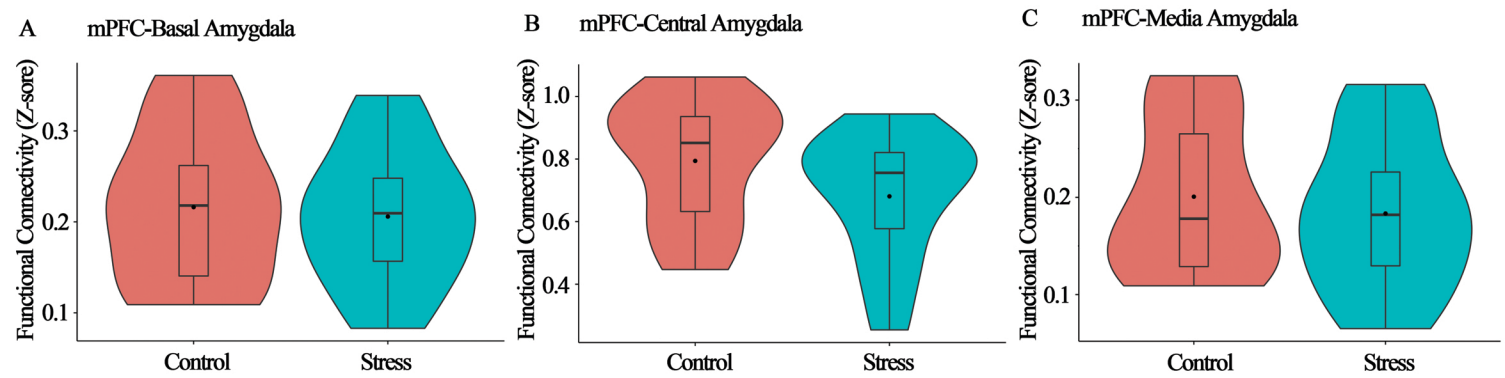

Supplement: Supplementary 1 — Figs. S1 to S4 [file research.0264.f1.zip › Figure S3.pdf]

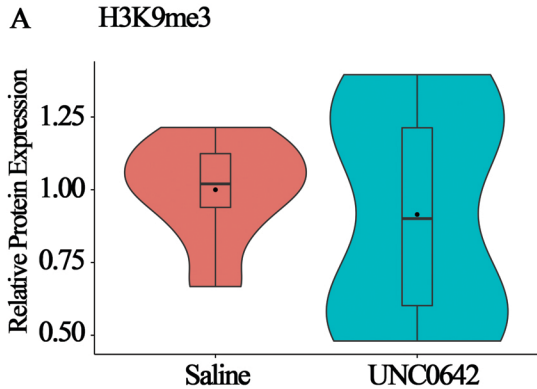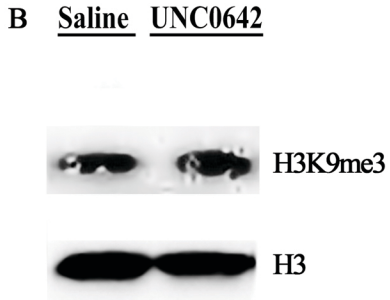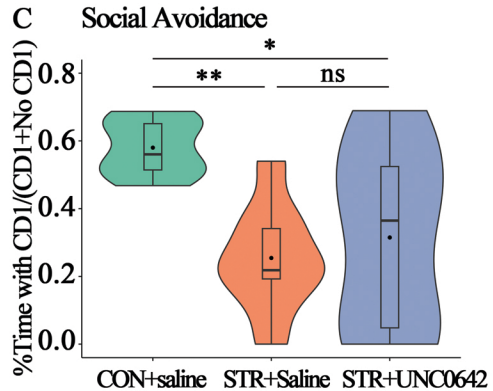

Supplement: Supplementary 1 — Figs. S1 to S4 [file research.0264.f1.zip › Figure S4.pdf]
